# Clinical features and nasal inflammation in asthma and allergic rhinitis

**DOI:** 10.1093/cei/uxac019

**Published:** 2022-02-18

**Authors:** Meiping Chen, Yijun Ge, Wanmi Lin, Haiping Ying, Wen Zhang, Xuechan Yu, Chunlin Li, Chao Cao

**Affiliations:** Department of Respiratory and Critical Care Medicine, Ningbo First Hospital, Ningbo, China; School of Medicine, Ningbo University, Ningbo, China; School of Medicine, Ningbo University, Ningbo, China; Department of Respiratory and Critical Care Medicine, Ningbo First Hospital, Ningbo, China; Department of Respiratory and Critical Care Medicine, Ningbo First Hospital, Ningbo, China; Department of Respiratory and Critical Care Medicine, Ningbo First Hospital, Ningbo, China; School of Medicine, Ningbo University, Ningbo, China; Department of Otorhinolaryngology-Head and Neck Surgery, Ningbo First Hospital, Ningbo, China; Department of Respiratory and Critical Care Medicine, Ningbo First Hospital, Ningbo, China

**Keywords:** asthma, allergic rhinitis, clinical features, nasal, inflammation

## Abstract

Asthma and allergic rhinitis (AR) are widely considered to be the most common chronic inflammatory disorders. This study was performed to investigate the clinical features, disease severity, and upper airway inflammation among patients with asthma, AR, and asthma comorbid AR. Blood and nasal lavage fluid samples were collected from patients with isolated asthma (*n* = 23), isolated AR (*n* = 22), and asthma comorbid AR (*n* = 22). Demographic data, symptom evaluation, and spirometry were obtained from all subjects. The levels of interleukin (IL)-4, IL-5, IL-13, IL-17, IL-25, IL-33, and S100 proteins were measured in the nasal lavage fluid. Compared with isolated asthma, patients with asthma comorbid AR showed a lower quality of life according to the asthma quality-of-life questionnaire (AQLQ) score (6.11 ± 0.47 vs. 6.45 ± 0.35*, P* = 0.007). Additionally, no significant difference in the levels of IL-4 (*P* = 0.116), IL-25 (*P* = 0.235), and S100A12 (*P* = 0.392) was observed in nasal lavage fluid among three groups. However, miniscule levels of IL-5, IL-17, IL-13, IL-33, S100A8, and S100A9 were detected in nasal lavage fluid in all three groups. Patients with asthma comorbid AR showed an increased level of systemic cytokine in plasma than that of patients with isolated AR or asthma alone. The finding from our study may help clinicians to better understand the airway inflammation among asthma patients with or without AR.

## Teaser

Compared with isolated asthma, patients with asthma comorbid AR showed a lower quality of life according to the asthma quality of life questionnaire (AQLQ) score. No significant difference in the levels of IL-4, IL-25, and S100A12 was observed in nasal lavage fluid among three groups. Patients with asthma comorbid AR showed an increased levels of systemic cytokine in plasma than that of patients with isolated AR or asthma alone.

## Graphical Abstract

**Graphical Abstract:**
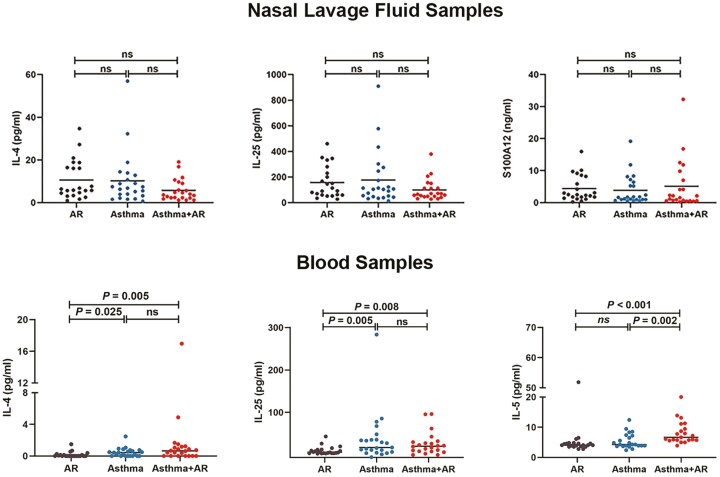

## Introduction

Asthma and allergic rhinitis (AR) are widely considered to be the most common chronic inflammatory disorders [1–3]. The prevalence of asthma and AR have been increasing rapidly, and have affected 1‒18% and 10‒40% of people around the world, respectively [1, 2, 4]. Grossman has proposed the concept of ‘one airway, one disease’ to explain the pathophysiology of asthma and AR [5]. In fact, AR coexisted in ~80% of asthmatics, and up to 40% of AR cases have reported asthma symptoms [6, 7]. Published evidence suggested a link between asthma and AR, and indicated that AR was one of the risk factors for asthma exacerbation [6, 8]. The comorbidity of upper and lower airways disease is often associated with poorly controlled asthma, affecting the quality of life, increasing the frequency of asthma-induced hospitalization, and raising healthcare-associated costs [3, 9].

Generally, asthma and AR are mainly induced by T helper type 2 (Th2 type) adaptive immune responses [10, 11]. Many studies have shown that type 2 cytokines, including interleukin (IL)-4, IL-5, and IL-13, were related to allergic inflammation and these cytokines elevated in the sputum and the blood of patients with asthma and AR [12–14]. Beside Th2-related cytokines, multiple studies have shown IL-25 and IL-33 were responsible for chronic inflammation [15]. Moreover, several S100 proteins, including S100A8, S100A9, and S100A12, were associated with inflammatory diseases, and have been reported to be elevated in the serum of patients with allergic disease [16, 17].

Although many studies have reported an association between asthma and AR, it is not clear whether nasal inflammation is present in patients with asthma. Additionally, whether the type 2 cytokines association holds at nasal inflammation in asthma and AR remains unexplored. Therefore, this study was conducted to investigate the clinical features, disease severity, and nasal inflammation among patients with asthma, AR, and asthma comorbid AR.

## Materials and Methods

### Subject groups and study design

This study was conducted in Ningbo First Hospital from August 2020 to February 2021. Patients were divided into three groups: isolated AR, isolated asthma, asthma comorbid AR. Asthma was diagnosed based on Global Initiative for Asthma (GINA) guidelines [18, 19]. AR was confirmed following the Allergic Rhinitis and its Impact on Asthma (ARIA) guideline [20]. The definition of asthma comorbid AR was also following ARIA guidelines [20]. Subjects were excluded if they met any of the exclusion criteria [1]: younger than 18 years or older than 80 [2]; pregnant or lactating females [3]; patients with chronic obstructive pulmonary disease (COPD) based on a post-bronchodilator the ratio of forced expiratory volume in first second (FEV_1_) to forced vital capacity (FVC) (FEV_1_/FVC) less than 0.7 predicted [4]; respiratory infections within past 4 weeks [5]; administered with corticosteroids orally or intravenously within previous 6 months [6]; exhibited acute asthma attack 8 weeks before the study.

Demographic data, symptoms, pulmonary function, and nasal lavage fluid were obtained from all subjects. This study was approved by Ningbo First Hospital Ethics Committee (approval 2020-R-145) and written informed consent was obtained from all participating subjects.

## Symptom scores

Total nasal symptom score (TNSS) was performed in patients with AR. Asthma Control Questionnaire (ACQ) [21] and Asthma Quality of Life Questionnaire (AQLQ) [22] were assessed for patients with asthma and patients with asthma comorbid AR, respectively. The AQLQ comprised five domains: limitation of activities, symptoms, emotional dysfunction, environment exposure, and self-health care. The scores were ranged from 1 to 7, and a lower score indicated more severe symptoms [23].

### Pulmonary function

Pulmonary function was measured using a computer-assisted spirometry (Jager, MasterScreen, Höchberg, Germany) according to the American Thoracic Society standards [24]. FEV_1_, peak expiratory flow (PEF), and forced expiratory flow between 25% and 75% of vital capacity (FEF (25–75)) were evaluated as the percentage of predicted. The ratio of FEV_1_/FVC was also recorded.

### Nasal lavage

Isotonic saline was brought to room temperature before being used for nasal lavage collection. Nasal lavage fluid samples were collected and the methods were referred to previous studies [25]. Each 20ml prefilled syringe was attached with a nasal olive properly. Subjects remained seated and kept their necks extended 30º in a forward-flexed neck position to avoid fluid from entering the nasopharynx. Lavage fluids (10 ml physiologic saline solution) were gently instilled into the nostril [26]. After 5 seconds, the samples were expelled into an appropriate sterile container. To ensure adequate washing, the isotonic saline was passed slowly in both middle meatus. Then, the fluids were collected in 15 ml conical tubes, and centrifuged at 3,000 rpm for 10 minutes at 4°C to remove cells and debris (Thermo Fisher, Waltham, MA, USA). The separated supernatant was placed in aliquots in 500 μl tubes, and stored at −80°C until analysis.

## Blood Samples

Blood samples were collected into ethylenediaminetetraacetic acid vacutainer tubes (New Jersey, BD, USA, 5 ml). Samples were immediately centrifuged for 10 minutes at 3,000 rpm at 4°C and the supernatant was separated in 200 µl tubes and stored at −80°C until analysis.

### Cytokine measurement

The levels of IL-5, IL-17, and IL-33 in the nasal lavage fluid were measured by enzyme-linked immunosorbent assay (ELISA) kits (R&D Systems, Minneapolis, MN, USA). The levels of IL-4, IL-13, IL-25, S100A8, S100A9, and S100A12 were analyzed by CUSABIO ELISA kits (CUSABIO, Wuhan, China). Blood samples of IL-4, IL-5, IL-25, and S100A12 were measured by standard quantitative ELISA kits (MultiSciences, Hangzhou, China; ElabScience, Wuhan, China; Proteintech, Wuhan, China; Abcam, Cambridge, UK). The lowest detection limit for each cytokine and chemokine were set as follows: R&D Systems: IL-5, 3.9 pg/ml; IL-33, 3.13 pg/ml; IL-17, 31.3 pg/ml. CUSABIO ELISA: IL-4, 6.25 pg/ml; IL-13, 62.5 pg/ml; IL-25, 62.5 pg/ml; S100A8, 1.25 ng/ml; S100A9, 4.69 ng/ml; S100A12, 0.01 ng/ml. MultiSciences: IL-4, 1.56 pg/ml. ElabScience: IL-25, 31.25 pg/ml. Proteintech: IL-5, 7.8 pg/ml; S100A12: 31.2 pg/ml. The optical density (OD) value was read and analyzed on a spectrophotometer (SoftMax Pro Version 7.1 software, Molecular Devices, USA). All assays were conducted according to the manufacturer’s protocols.

### Statistical analysis

Results were expressed as mean and standard deviation (SD) unless specified otherwise. Mann–Whitney U tests or two independent sample *t*-tests were employed for continuous variables, and χ^2^ tests were used to compare categorical variables between groups. Kruskal–Wallis rank tests were used to evaluate differences among three groups. All correlations were performed with Spearman rank-sum tests. *P*-values less than 0.05 were considered as significant. Statistical analysis was conducted with GraphPad Prism 8 (GraphPad Software, San Diego, CA, USA), and SPSS 21.0 (SPSS Inc., Chicago, IL, USA).

## Results

### Subjects

Sixty-seven patients with diseases were included in this study. Details of the demographic characteristics are reported in Table 1. Briefly, 22 patients with isolated AR consisted of 10 males and 12 females, and the mean age was 41.0 years old; 23 patients with isolated asthma consisted of 13 males and 10 females, with a mean age of 41.6 years old; 22 patients with asthma comorbid AR consisted of 10 males and 12 females, and their age was 40.0 years old in average. No statistically difference was observed among three groups, with regards to gender (*P* = 0.713), age (*P* = 0.904), smoking status (*P* = 0.618), body mass index (BMI) (*P* = 0.435), and atopic history (*P* = 0.148; Table 1).

**Table 1:** Demographic data of study participants

Characteristics	Patients with allergic diseases			*P*-Value
	AR	Asthma	Asthma + AR	
Subjects	22	23	22	
Age, year	40.95 ± 10.52	41.57 ± 13.81	40.00 ± 12.17	0.904
Sex, M/F	10/12	13/10	10/12	0.713
Smoking status				
Never	16 (72.73)	19 (82.61)	16 (72.73)	0.618
Past	4 (18.18)	1 (4.35)	4 (18.18)	
Current	2 (9.09)	3 (13.04)	2 (9.09)	
BMI, kg/m^2^	24.21 ± 2.20	22.95 ± 3.45	23.99 ± 2.50	0.435
Atopic history	10 (45.45)	8 (34.78)	14 (63.64)	0.148
FEV_1_, %predicted	103.61 ± 12.40	81.70 ± 19.76	85.66 ± 20.59	<0.001
FEV_1_/FVC, %	87.13 ± 7.37	75.39 ± 9.38	77.04 ± 11.93	<0.001
PEF, %	95.44 ± 10.28	87.97 ± 26.42	90.05 ± 21.90	0.468
FEF (25–75), %	92.42 ± 21.37	52.00 ± 24.62	63.00 ± 34.64	<0.001
ACQ score	NA	1.19 ± 0.83	1.16 ± 0.81	0.901
AQLQ score				
Total score	NA	6.45 ± 0.35	6.11 ± 0.47	0.007
Limitation of activities	NA	6.35 ± 0.54	5.76 ± 0.61	0.001
Asthma symptoms	NA	6.23 ± 0.57	6.10 ± 0.83	0.537
Emotional dysfunction	NA	6.77 ± 0.34	6.50 ± 0.47	0.028
Environment exposure	NA	6.75 ± 0.47	6.51 ± 0.76	0.329
Self-health care	NA	6.49 ± 0.62	6.17 ± 0.80	0.141

AR, allergic rhinitis, no asthma; Asthma + AR, asthma comorbid AR. Values were expressed as mean ± SD or n (%) unless otherwise noted. BMI, body mass index; FEV_1_, forced expiratory volume in first second; FVC, forced vital capacity; PEF, peak expiratory flow; FEF (25–75), forced expiratory flow between 25% and 75% of vital capacity; ACQ, the Asthma Control Questionnaire; AQLQ, the Asthma Quality of Life Questionnaire; NA, not applicable.

FEV_1_, %predicted: AR: asthma. *P* < 0.001; asthma: asthma + AR, *P* = 0.001.

FEV_1_/FVC, %: AR: asthma. *P* < 0.001; asthma: asthma + AR, *P* = 0.002.

FEF (25–75), %: AR: Asthma. *P* < 0.001; Asthma: Asthma + AR, *P* = 0.002.

### Clinical features

**Figure 1:**
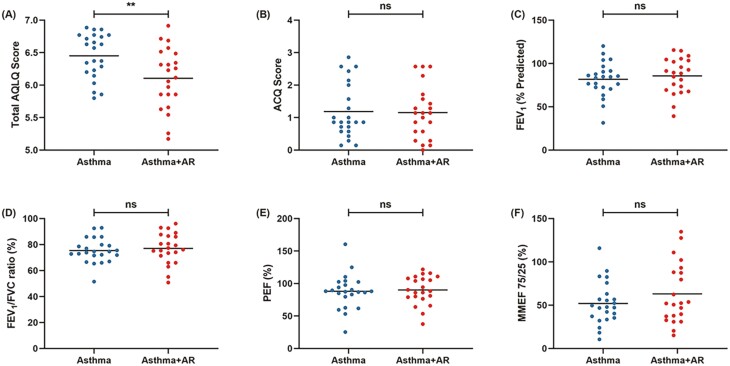
Clinical characteristics of AQLQ (A), ACQ (B), FEV_1_ (% predicted) (C), FEV_1_/FVC (D), PEF (E), and FEF (25–75) (F) in patients with isolated asthma and asthma comorbid AR. Mean values were shown as horizontal bars. *P* < 0.05 was considered as statistically significant, *P < 0.05, **P < 0.01. AR, allergic rhinitis; Asthma + AR, asthma comorbid AR; ACQ, Asthma Control Questionnaire; AQLQ, Asthma Quality of Life Questionnaire; FEV_1,_ forced expiratory volume per second; PEF, peak expiratory flow; FEF (25–75), forced expiratory flow between 25% and 75% of vital capacity; ns, not significant.

Compared with those with isolated asthma, patients with asthma comorbid AR showed a lower total AQLQ score (6.11 ± 0.47 *vs.* 6.45 ± 0.35; *P* = 0.007; Fig. 1A) and a diminished physical activity score (5.76 ± 0.61 *vs.* 6.35 ± 0.54; *P* = 0.001; Table 1), suggesting a lower quality of life and more severe physical limitations in patients with asthma comorbid AR. Similarly, a lower score for emotional dysfunction was also observed in patients with asthma comorbid AR compared with isolated asthma (6.50 ± 0.47 vs. 6.77 ± 0.34; *P* = 0.028; Table 1). However, no significant difference was found in the score of asthma symptoms (*P* = 0.537), environment exposure (*P* = 0.329), or self-health care (*P* = 0.141) between patients with isolated asthma and asthma comorbid AR (Table 1). ACQ score was similar in patients with isolated asthma and asthma comorbid AR (1.19 ± 0.83 *vs.* 1.16 ± 0.81; *P* = 0.901; Fig. 1B).

Pulmonary function was performed in all subjects. The values of predicted FEV_1_ were lower in patients with isolated asthma (mean ± SD; 81.70 ± 19.76%) compared with the patients with isolated AR (103.61 ± 12.40%; *P* < 0.001; Table 1). Lower values of FEV_1_/FVC ratio were also found in patients with isolated asthma (75.39 ± 9.38%), in comparison with isolated AR (87.13 ± 7.37%; *P* < 0.001; Table 1). Similarly, the values of FEF (25–75) were significantly reduced in patients with isolated asthma (52.00 ± 24.62%) than isolated AR (92.42 ± 21.37%; *P* < 0.001; Table 1). However, no significant difference was found in pulmonary function in patients with isolated asthma and asthma comorbid AR, including predicted FEV_1_ (*P* = 0.514), FEV_1_/FVC ratio (*P* = 0.607), PEF (*P* = 0.776), and FEF (25–75) (*P* = 0.229; Fig. 1 C‒F)

### Nasal inflammation

**Figure 2:**
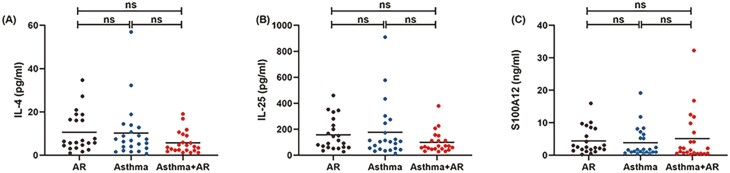
Levels of IL-4, IL-25, and S100A12 in the nasal lavage fluid of patients with isolated AR, isolated asthma, and asthma comorbid AR. No significant difference was found in IL-4 (A), IL-25 (B), or S100A12 (C) between groups. Median values were shown as horizontal bars and *P < 0.05 was considered as statistically significant. AR, allergic rhinitis; Asthma + AR, asthma comorbid AR; ns, not significant.

The levels of chemokines and cytokines in nasal lavage fluid were shown in Fig. 2. No significant difference in the level of IL-4 was observed in nasal lavage fluid among patients with isolated AR (median [interquartile range (IQR)], 6.09 pg/ml [4.17‒17.02 pg/ml]), isolated asthma (6.75 pg/ml [3.20‒12.80 pg/ml]), and asthma comorbid AR (3.78 pg/ml [2.23‒9.18 pg/ml]; *P* = 0.116; Fig. 2A). Likewise, there was no apparent difference in the levels of IL-25 in nasal lavage fluid among patients with isolated AR (102.84 pg/ml [59.36‒242.14 pg/ml]), isolated asthma (103.95 pg/ml [46.68‒246.15 pg/ml]) and asthma comorbid AR (67.26 pg/ml [47.73‒121.89 pg/ml], *P* = 0.235, Fig. 2B). Moreover, patients with isolated AR (2.49 ng/ml [1.65‒8.28 ng/ml]) did not differ significantly from isolated asthma (1.70 ng/ml [0.94‒6.40 ng/ml]), or patients with asthma comorbid AR (1.81 ng/ml [0.55‒7.70 ng/ml; *P* = 0.392, Fig. 2C]) in the levels of S100A12. Very low levels of IL-5, IL-17, IL-13, IL-33, S100A8, and S100A9 were detected in nasal lavage fluid in all three groups. In total, 66 patients (98.51%) were below the lower limit of quantification (LoD) of IL-5 (3.9 pg/ml) and S100A8 (1.25 ng/ml), 65 patients (97.01%) were below the LoD of IL-13 (62.5 pg/ml) and S100A9 (4.69 ng/ml); and 67 patients (100.00%) were below the LoD of IL-17 (31.3 pg/ml) and IL-33 (3.13 pg/ml).

**Figure 3:**
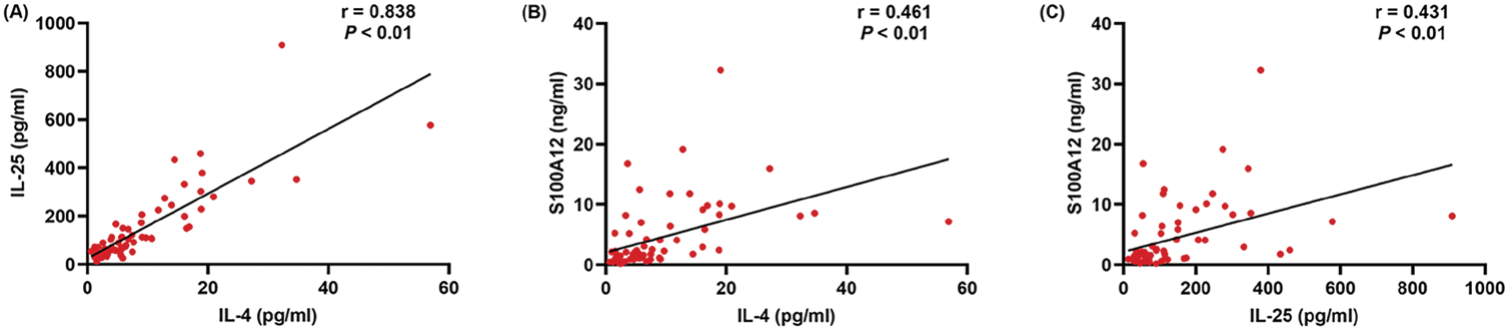
Correlations between IL-4 and IL-25 (A), IL-4 and S100A12 (B), IL-25 and S100A12 (C) in the nasal lavage fluid of patients with isolated AR, isolated asthma, and asthma comorbid AR. *P < 0.05 was considered as statistically significant. AR, allergic rhinitis; Asthma + AR, asthma comorbid AR.

**Figure 4:**
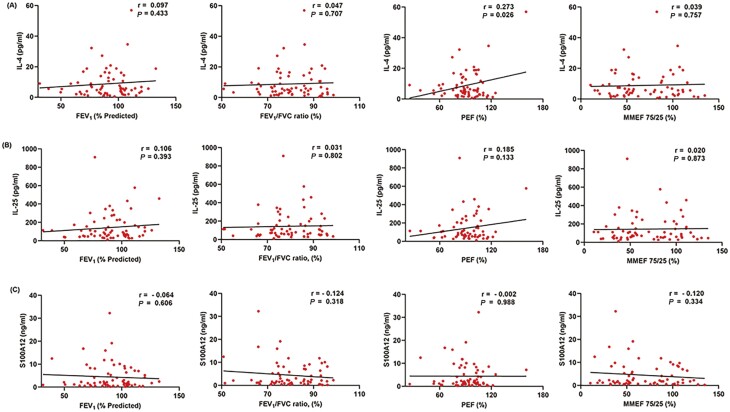
Correlations between pulmonary function and IL-4 (A), pulmonary function and IL-25 (B), pulmonary function and S100A12 (C) in the nasal lavage fluid of patients with isolated AR, isolated asthma, and asthma comorbid AR. *P < 0.05 was considered as statistically significant. AR, allergic rhinitis; Asthma + AR, asthma comorbid AR.

To explore the relationship among the levels of IL-4, IL-25, and S100A12 in nasal lavage fluid in patients with AR, asthma, and asthma comorbid AR, we performed Spearman correlation coefficient tests. As shown in Fig. 3A, strong positive correlations were observed between the levels of IL-4 and IL-25 in nasal lavage fluid (*r* = 0.838, *P* < 0.0001, Fig. 3A). In addition, the levels of IL-4 positively correlated to S100A12 (*r* = 0.461, *P* < 0.0001, Fig. 3B). Moreover, significant positive correlation was observed between the levels of IL-25 and S100A12 (*r* = 0.431, *P* < 0.001, Fig. 3C). Spearman correlation analysis was also conducted to further investigate the association between pulmonary function and the levels of IL-4, IL-25, S100A12 in nasal lavage fluid, yet, no significant correlation was observed (Fig. 4).

Patients with asthma comorbid AR exhibited higher plasma levels of cytokines IL-4 and IL-25 than those without asthma, respectively (IL-4, *P* = 0.025, Supplementary Fig. 1; IL-5, *P* = 0.005, Supplementary Fig. 2). However, IL-4 and IL-25 were similar in asthma patients with or without comorbid AR. In line with plasma IL-4 and IL-25 data, patients with asthma comorbid AR had higher levels of IL-5 (6.94 [5.74, 10.69]) than those without asthma (4.07 pg/ml [3.61, 4.72] pg/ml, *P* < 0.001). Plasma S100A12 was below the detection limit in all three groups.

## Discussion

Although previous studies suggested the concept of ‘one airway, one disease’ between asthma and AR, there were little data regarding the levels of nasal inflammation among patients with AR, asthma, and asthma comorbid AR [27–29]. Therefore, we performed this study to investigate clinical features and nasal inflammation among patients with isolated AR, isolated asthma, and asthma comorbid AR. Interestingly, in this study, we observed a similar degree of Th2-type inflammation in the nasal lavage fluid among all three groups of patients. Hoverer, asthma comorbid AR patients showed an increase in the expression of systemic cytokine (IL-4, IL-5, and IL-25) in plasma; this was associated with a lower quality of life than that of patients with asthma. These results suggest that asthma comorbid AR patients may be more likely to develop more potent systemic inflammation than those with isolated AR or asthma alone. The findings from our study support the concept of ‘one airway, one disease’ in upper airway inflammation. In addition, there were other factors, other than upper airway inflammation, that affected the quality life of asthma comorbid AR patients.

Asthma and AR are common Th2-type inflammation airway diseases. Th2-type-mediated production of cytokines played a key role in enhanced and sustained activation in chronic allergic inflammatory disease [30–32]. Previous data reported various cytokines, such as IL-4, IL-5, IL-13, and IL-33, increased in patients with asthma and AR in serum or lower respiratory tract specimens [26, 33–35]. However, fewer studies were conducted to investigate upper airway inflammation in asthma patients, especially in those with asthma comorbid AR.

IL-4 and IL-13 were recognized as the central regulators in allergic inflammation, promoting IgE class switching and mast cell activation [3, 36]. In addition, IL-25 and IL-33 also played crucial roles in the development of chronic inflammation [15]. There is evidence that IL-25 and IL-33 released by damage mucosal epithelial cells mainly contributed to innate and adaptive immune responses, and primarily promoted a type-2 immune response [37, 38]. Eifan *et al.* found a higher levels of IL-4, IL-5, and IL-13 in nasal lavage fluid in patients with AR than that of healthy controls [26]. In another report, the authors also observed increased levels of IL-5 in nasal lavage fluid in seasonally AR subjects [39]. In this study, multiple Th2 type cytokines were detected in nasal lavage fluid among patients with AR, isolated asthma, and asthma comorbid AR. No significant difference in the levels of IL-4 or IL-25 was observed in nasal lavage fluid among the three groups. However, very low levels of IL-5, IL-17, IL-13, IL-33 were detected in nasal lavage fluid in all three groups.

S100 family of members (S100s), known as the calcium (Ca^2+^)-binding proteins, have been reported to involve in the regulation of allergic inflammation [40]. S100A8, S100A9, and S100A12 markedly elevated in inflammatory diseases and induced innate inflammatory responses via the activation of Toll-like receptor 4 (TLR4) [17]. S100A8, S100A9, and S100A12 were supposed to directly stimulate the production of mucin 5AC (MUC5AC), a major mucin protein in the airway which closely correlated with airway inflammation [16]. In this study, similar levels of S100A12 in nasal lavage fluid were found among the three groups. Interestingly, a significant positive correlation was found among the levels of IL-4, IL-25, and S100A12 in nasal lavage fluid. These data may suggest S100A12 involved in airway inflammation and possibly related to IL-4 and IL-25. However, neither the levels of S100A8 nor S100A9 were below the LoD in nasal lavage fluid in all three groups.

In this study, no statistical difference in pulmonary function was found between patients with isolated asthma and asthma with comorbid AR. However, compared with isolated asthma, patients with asthma and comorbid AR showed a lower quality of life according to the AQLQ score, especially in physical limitation and emotional state. It should be noted that a similar degree of upper airway inflammation was observed between patients with asthma and asthma comorbid AR, despite increases in plasma cytokine levels in patients with asthma comorbid AR. The results show more severe and extensive inflammation in subjects with asthma comorbid AR than in those with isolated AR or asthma alone. Furthermore, a possible explanation for low quality of life in asthma comorbid AR patients was rhinitis-associated symptoms, including sneeze, nasal congestion, nasal leakage, and itching-caused disorders of work and life [41]. The results from this study suggested that physicians should pay more attention to improve the quality of life in patients with asthma and AR comorbidities.

Some limitations of this study should be acknowledged. First, this study investigated Th2-type inflammation in three allergic diseases, while healthy controls were not enrolled. Second, very low concentrations of several cytokines measured by ELISA in nasal lavage fluid, more sensitive methods are needed in future studies. Even though, our study has some advantages. To the best of our knowledge, this is the most comprehensive study to date to quantitatively evaluate a series of Th2-type cytokines in nasal lavage fluid. In addition, the findings from our study suggest a similar degree of Th2-type inflammation in the nasal lavage fluid between patients with asthma and asthma comorbid AR, but asthma comorbid AR had a lower quality of life than that of patients with asthma. Such evidence might help physicians and patients to have a better understanding of the relationship between airway inflammation and disease control in asthma patients with or without AR.

## Conclusion

In summary, patients with asthma, AR, and asthma comorbid AR shared a similar degree of Th2-type inflammation in the nasal lavage fluid. These findings supported the concept of ‘one airway, one disease’. However, patients with asthma comorbid AR showed an increased expression of systemic cytokines in plasma, and these patients had a reduced quality of life compared with those with isolated asthma. This knowledge would be of great help for clinicians to better understand and pay attention to improve quality of life in asthma comorbid AR patients.

## Supplementary Material

uxac019_suppl_Supplementary_Material

## Data Availability

All data included in this study are available upon request by contact with the corresponding author.

## Abbreviations

ACQ: asthma control questionnaire
AQLQ: Asthma quality-of-Life Questionnaire
AR: allergic rhinitis
ARIA: allergic rhinitis and its impact on asthma
BMI: body mass index
Ca: calcium
COPD: chronic obstructive pulmonary disease
ELISA: enzyme-linked immunosorbent assay
FEF (25–75): FORCED expiratory flow between 25% and 75% of vital capacity
FEV1: forced expiratory volume in first second
GINA: Global Initiative for Asthma
IL: interleukin
MUC5AC: mucin 5AC
NA: not applicable
OD: optical density
PEF: peak expiratory flow
SD: standard deviation
S100s: S100 family of members
Th2 type: T-helper type 2
TLR4: Toll-like receptor 4
TNSS: total nasal symptom score
