# Correction to: Clinical features and nasal inflammation in asthma and allergic rhinitis

**DOI:** 10.1093/cei/uxac036

**Published:** 2022-05-10

This is a correction to: Meiping Chen, Yijun Ge, Wanmi Lin, Haiping Ying, Wen Zhang, Xuechan Yu, Chunlin Li, Chao Cao, Clinical features and nasal inflammation in asthma and allergic rhinitis, *Clinical and Experimental Immunology*, 2022, uxac019, https://doi.org/10.1093/cei/uxac019

In the originally published version of this manuscript, the affiliation indicators were out of order. The correct affiliations are as follows:

Meiping Chen^1,2^, Yijun Ge^1,2^, Wanmi Lin^1^, Haiping Ying^1^, Wen Zhang^1^, Xuechan Yu^2^, Chunlin Li^3,*^ and Chao Cao^1,*^

^1^Department of Respiratory and Critical Care Medicine, Ningbo First Hospital, Ningbo, China

^2^School of Medicine, Ningbo University, Ningbo, China

^3^Department of Otorhinolaryngology-Head and Neck Surgery, Ningbo First Hospital, Ningbo, China

This error has been corrected (online).

