# Supplementary material for: Clinical features and nasal inflammation in asthma and allergic rhinitis
Source: Clin Exp Immunol. 2022 Feb 18;208(1):25–32. doi: 10.1093/cei/uxac019 (PMC9113297; doi:10.1093/cei/uxac019)

**Clinical features and nasal inflammation in asthma and allergic rhinitis**

**Running title: Nasal inflammation in asthma and allergic rhinitis**

**Online Supplement**

Meiping Chen, Yijun Ge, Wanmi Lin, Haiping Ying, Wen Zhang, Xuechan Yu, Chunlin Li, Chao Cao

**eFigure legends**

**eFigure 1.** Plasma levels of IL-4 in the patients with isolated AR, isolated asthma, and asthma comorbid AR. Median values were shown as horizontal bars and *P* < 0.05 was considered as statistically significant. AR, allergic rhinitis; Asthma + AR, asthma comorbid AR; ns, not significant.

**eFigure 2.** Plasma levels of IL-25 in the patients with isolated AR, isolated asthma, and asthma comorbid AR. Median values were shown as horizontal bars and *P* < 0.05 was considered as statistically significant.

**eFigure 3.** Plasma levels of IL-5 in the patients with isolated AR, isolated asthma, and asthma comorbid AR. Median values were shown as horizontal bars and *P* < 0.05 was considered as statistically significant.

**SUPPLEMENTARY FIGURES**

**eFigure 1**


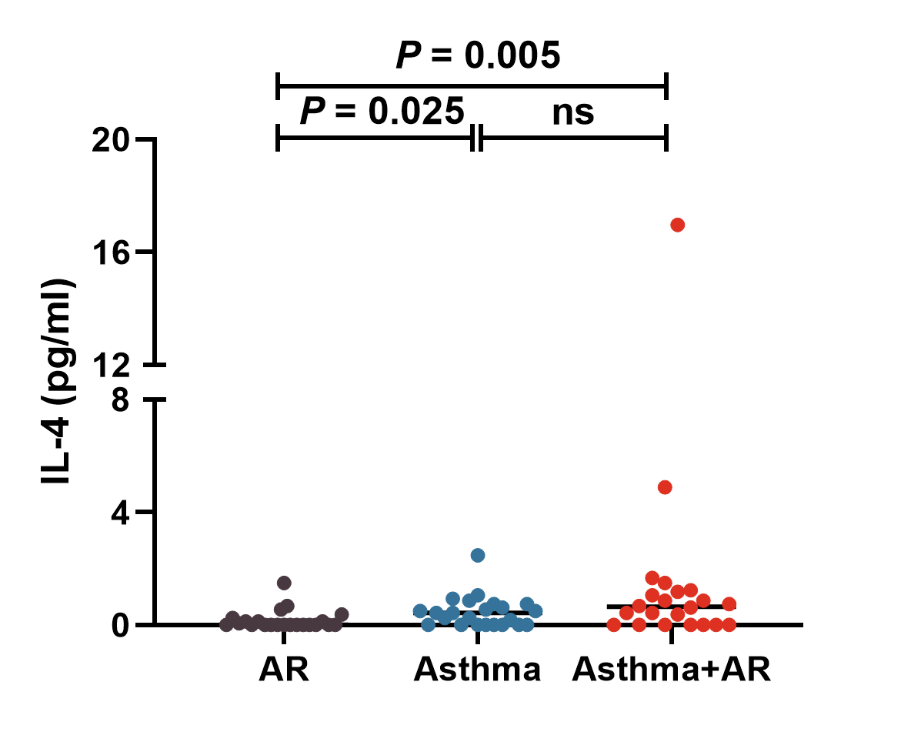


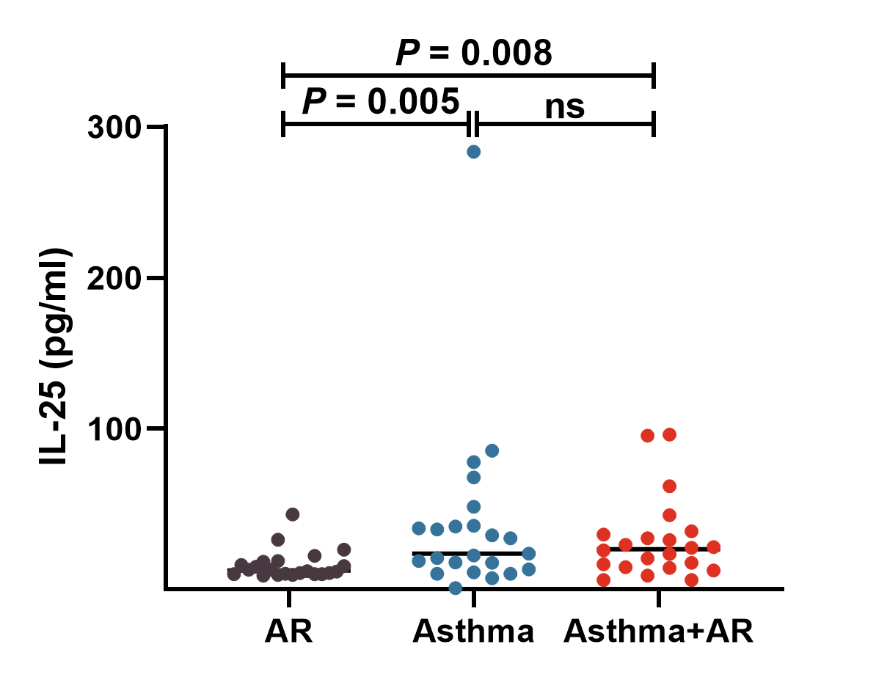
 **eFigure 2**

**eFigure 3**


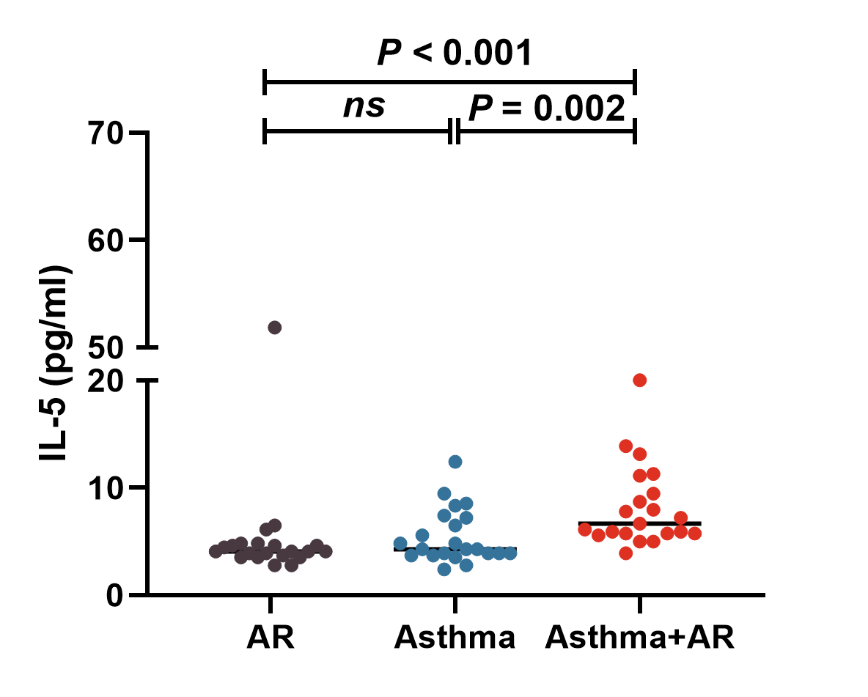

Supplement: uxac019_suppl_Supplementary_Material [file uxac019_suppl_supplementary_material.docx]
